# Refractory ventricular tachycardia and heart failure due to anti-mitochondrial antibody-positive inflammatory myopathy

**DOI:** 10.1186/s12872-023-03057-6

**Published:** 2023-01-31

**Authors:** Rong Huang, Xinlin Zhang, Zhonglin Han, Xiang Wu, Guannan Li, Jianzhou Chen, Biao Xu, Rong Gu, Lian Wang

**Affiliations:** Department of Cardiology, Nanjing Drum Tower Hospital, The Affiliated Hospital of Nanjing University Medical School, Nanjing, China

**Keywords:** Anti-mitochondrial antibody-positive inflammatory myopathy, Ventricular tachycardia, Heart failure, Case report

## Abstract

**Background:**

Anti-mitochondrial antibody (AMA)-positive inflammatory myopathy, a rare type of idiopathic inflammatory myopathy which was frequently difficult to diagnose, can affect muscles and the structure and electrical conduction of the heart. Early identification and treatment of this myopathy can prevent serious cardiovascular adverse events and improve cardiac function.

**Case presentation:**

We report a patient who experienced repeated syncope, ventricular tachycardia (VT) and heart failure accompanied by weakness and muscle atrophy. He was initially diagnosed with dilated cardiomyopathy and received implantable cardioverter-defibrillator therapy. He was subsequently misdiagnosed as muscular dystrophy due to progressive muscular atrophy. However, the patient developed repeated and refractory VT storms that were not alleviated by conventional therapy. Finally, he was diagnosed with AMA-positive inflammatory myopathy with cardiac injuries. The patient was markedly recovered by being treated with immunosuppressive and immunomodulatory therapy.

**Conclusion:**

AMA could be screened when discovering myopathies accompanied by unexplained cardiac symptoms. Our findings provide insights into the diagnosis and therapy of this rare and severe disease.

## Background

Anti-mitochondrial antibody (AMA)-positive inflammatory myopathy is a rare type of idiopathic inflammatory myopathy. Not only affects muscles, but also the structure and electrical conduction of the heart. The myositis was frequently difficult to diagnose, and the clinical manifestations of it were various, mild or severe. We report the case of a patient with AMA-positive inflammatory myopathy who experienced severe cardiac complications and the muscles of eye and limbs involvement.

## Case presentation

**Fig. 1:**
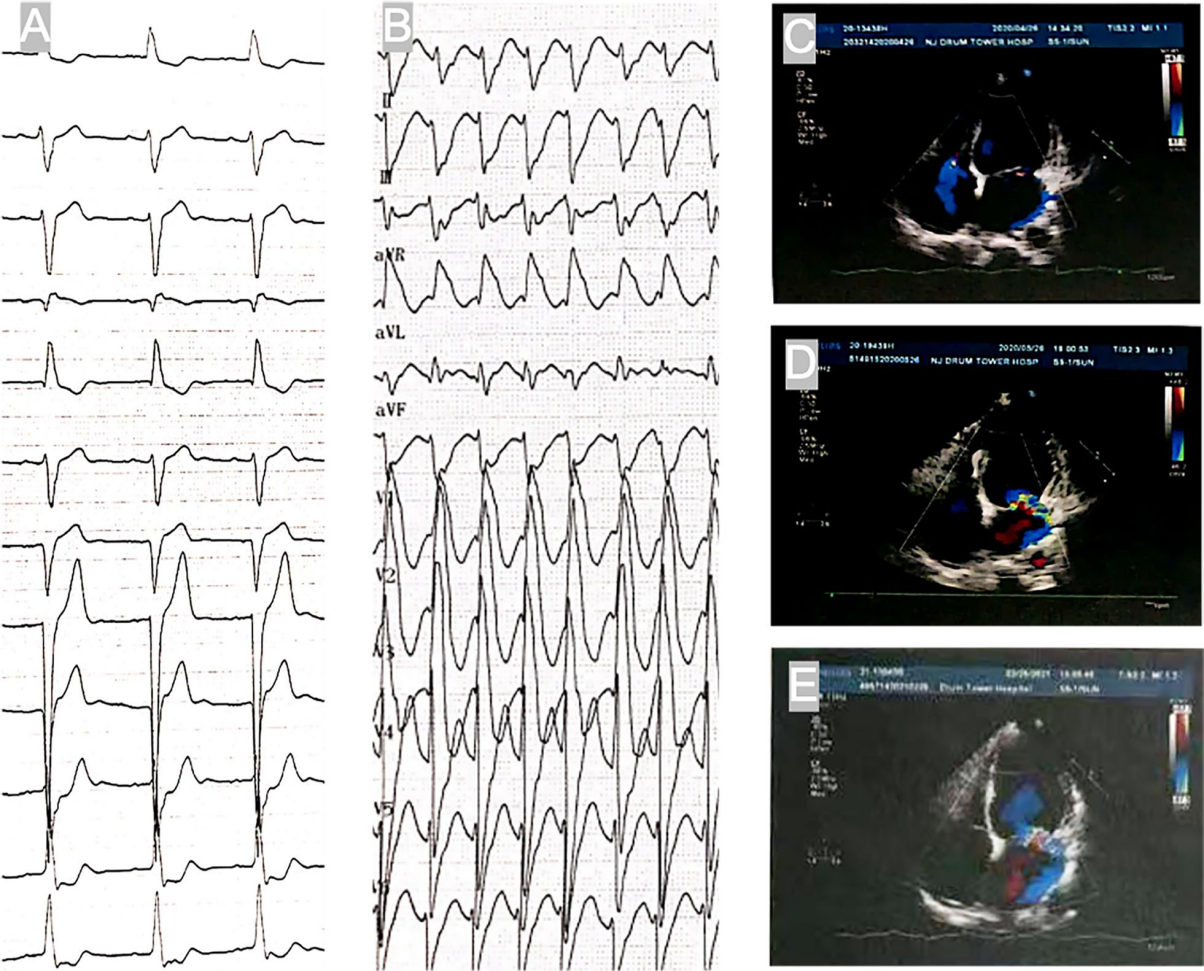
The ECG evolution and Echocardiography during hospitalization. **A** Sinus rhythm with HR of 74 bpm after VT discontinued. **B** Sustained VT with HR of 166 bpm when admission. **C** Enlarged chambers (LVDd 64 mm) and LVEF 28% on echocardiography. **D** Cardiac remodeling was reversed after the therapy (LVEF 33%, LVDd 60 mm). **E** Remarkable recovery in last follow-up on echocardiography (LVEF 52%, LVDd 59 mm)

A 37-year-old male patient was admitted to a local general hospital in October 2019 due to persistent fatigue for 1 year and repeated syncope for 1 month. Physical examination showed marked atrophy of the muscles of the extremities. The electrocardiogram (ECG) captured ventricular tachycardia (VT) with heart rate (HR) of 145 bpm (Fig. 1A). The blood tests revealed creatine kinase (CK) 1067 U/L (reference range: 20–174 U/L) and creatine kinase isoenzyme (CK-MB) 112 U/L (reference range: 0–18 U/L) respectively. Cardiac magnetic resonance (CMR) imaging revealed that a possibility of atypical myocarditis. Computed tomography angiography (CTA) showed that the coronary and intracranial arteries were normal. Echocardiography demonstrated enlargement of left and right chambers (LVDD 57 mm) with a reduced left ventricular ejection fraction (LVEF 42%). The above tests implied that the pathological VT was probably associated with neuromuscular or immune disease. Nonetheless, the patient rejected the further tests, and received implantable cardioverter-defibrillator (ICD) therapy. 5 mg of bisoprolol once daily was given for long-term use. The patient had a history of chronic hepatitis B and no other disease.

In November 2019, the patient was hospitalized in the department of neurology of our hospital for aggravating muscle atrophy. Electromyogram (EMG) showed active myogenic damage. Muscle biopsy of the right biceps brachii demonstrated sporadic necrotic fibers with regeneration, but without obvious inflammatory cell infiltration. Autoimmune antibodies were measured and positive for anti-nuclear antibodies (ANA) and AMA. The muscular dystrophy was considered and coenzyme Q10 was prescribed in addition to the previous medications.

**Fig. 2:**
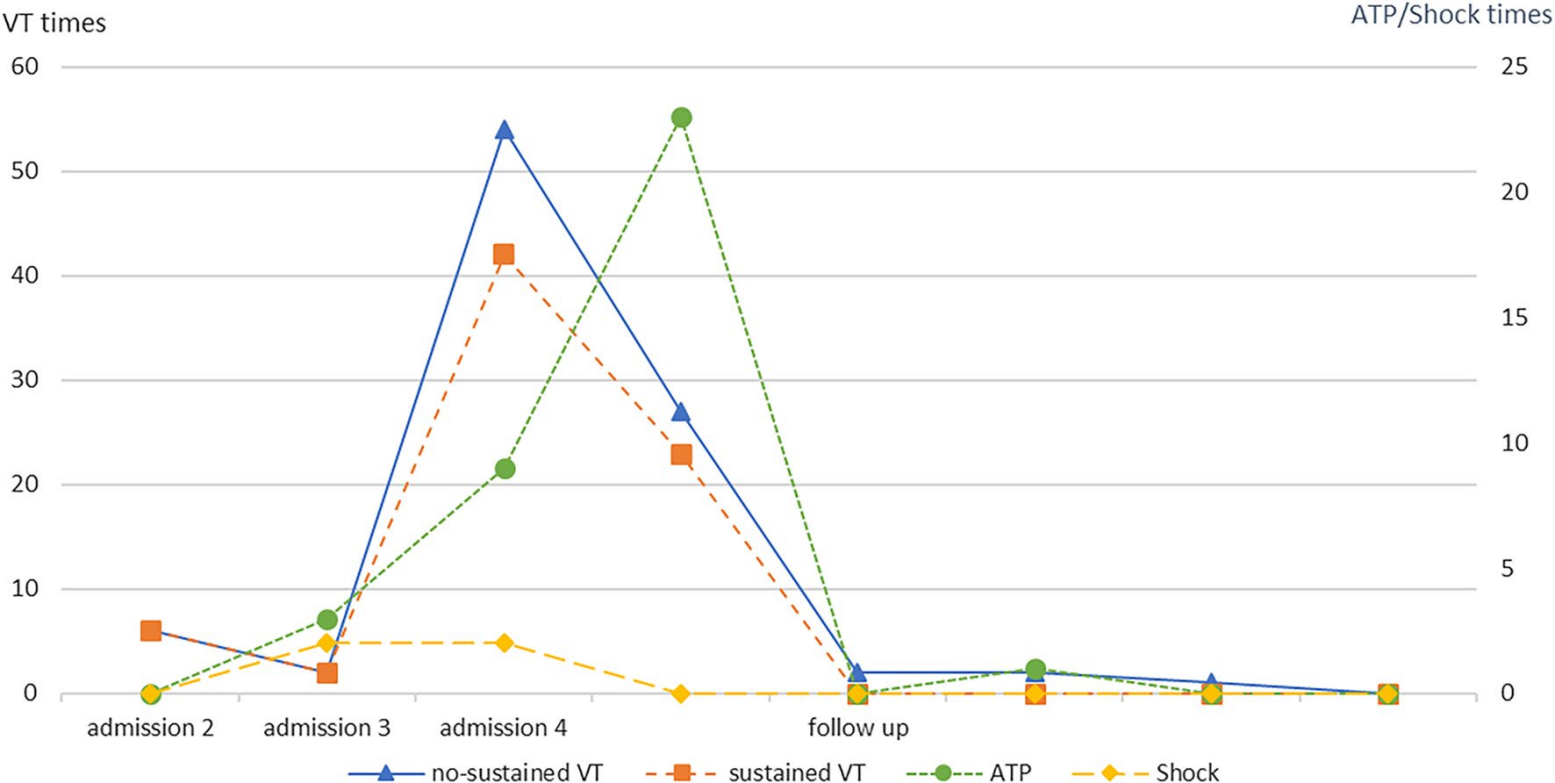
Timeline of VT and ATP treatment

In April 2020, the VT recurred and could terminate spontaneously (Fig. 2). The patient was hospitalized again. He was administrated amiodarone (200 mg/day) and methylprednisolone (24 mg/day). The VT did not come out thereafter.

Unfortunately, the patient suffered from repeated sustained VT in May 2020 (Fig. 2). According to ICD programming, since the day before admission, the patient had 43 VT, with a HR of 180 ± 5 bpm, and received 37 ATP treatments and 1 discharge treatment (Fig. 2). Meanwhile, he had new onset of blurred vision and diplopia.

The vital signs: T: 36.2 °C , P: 70 beats/min, R: 18/min, BP: 97/60mmHg. The strength of proximal limb muscle was grade 3 out of 5, the distal limb muscle was grade 4 and the bilateral tendon reflex was active.

Laboratory tests showed Troponin T 0.098 ug/L (reference range: 0.020–0.130 ug/L), CK 163 U/L, CK-MB 17 U/L, brain natriuretic peptide (BNP) 178 pg/mL (reference range: <100 pg/mL), glutamic-pyruvic transaminase 51.2 U/L (reference range: 0–40 U/L). The renal function and coagulation function were nearly normal. ECG showed VT with HR of 166 bpm (Fig. 1B). Echocardiography revealed that LVEF decreased to 28% and LVDD increased to 64 mm, Regional wall motion abnormality (RWMA) was not detected (Fig. 1C).

Upon admission, esmolol, dexmedetomidine and amiodarone were administered to control the VT storm and under sedation. Besides, sacubitril/valsartan (50 mg/day), bisoprolol (7.5 mg/day), spironolactone (20 mg/day), and entecavir (0.5 mg/day) were also administered. However, these measurements did not work.

**Fig. 3:**
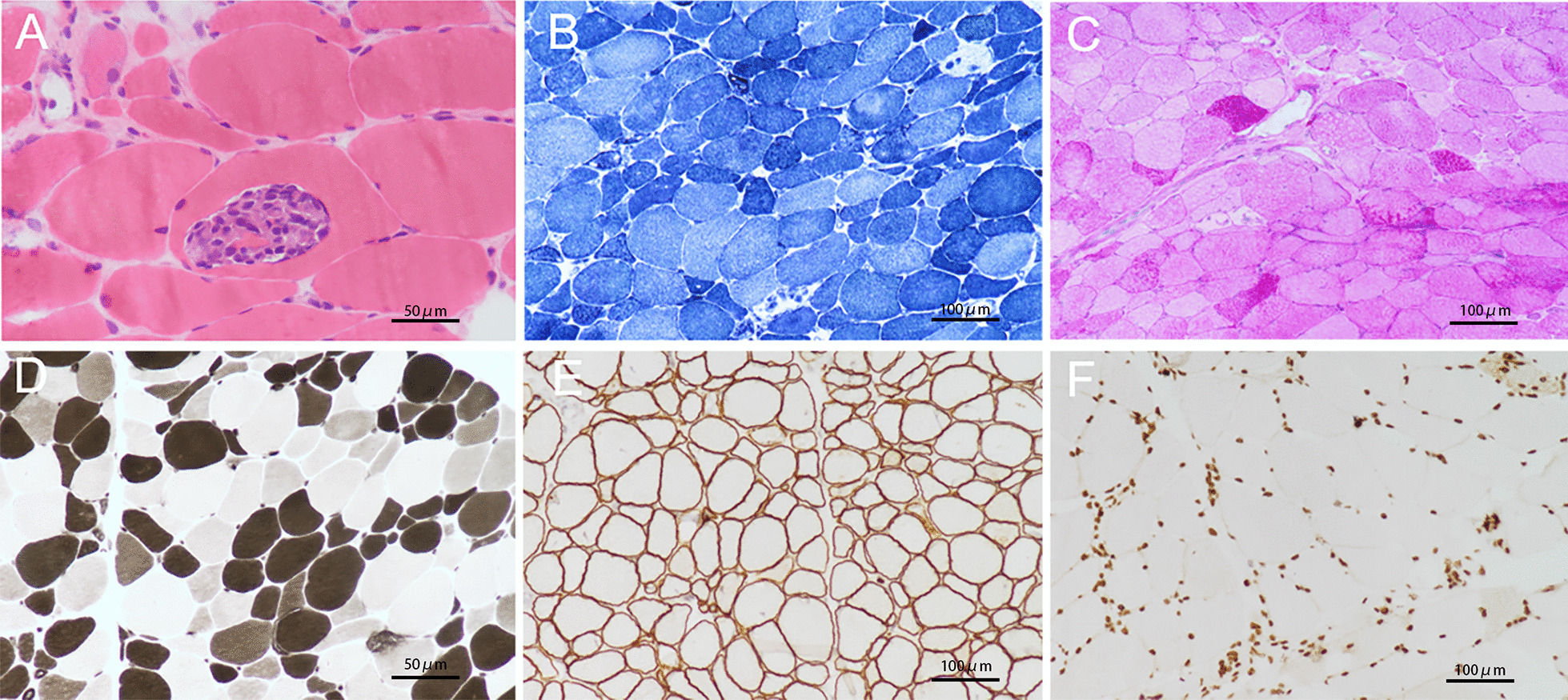
Histopathologic findings of Muscle biopsy. **A** Muscle fibers size is variable, and the muscle fibers are split (hematoxylin and eosin stain, ×400). **B** Worm-eaten fiber and change of mitochondrial enzyme activity (nicotinamide adenine dinucleotide stain ×200). **C** Type 1 muscle fiber glycogen slightly increased (periodic acid-schiff stain, ×200). **D** Muscle fibers are affected (ATPase 4.6 stain, ×400). **E** The expression of sarcoglycan was normal (immunohistochemical stain, ×200). **F** The expression of emerin was normal (immunohistochemical stain, ×200). Type of equipment: microscope: OLYMPUS BX53 LED; Acquisition software: OlympusCellSens

In order to identify the aetiology, the previous histologic slice was overdyed. The results showed some muscle fibers atrophy and a few denatured and necrotic muscle fibers (Fig. 3A–D). Combined with negative genetic testing results and immunohistochemical stain, muscular dystrophy and mitochondrial encephalomyopathy can be ruled out (Fig. 3E, F). According to the advice of the rheumatologist, the patient was diagnosed as AMA-positive idiopathic inflammatory myopathy (IIM). Furthermore, Immune-mediated necrotizing myopathy (IMNM) could be confirmed according to 2017 EULAR-ACR classification criteria [1]. Therefore, all the aforementioned symptoms and clinical signs were associated with the myositis.

Thereafter, he received intravenous methylprednisolone 40–80 mg/day for 5 days, intravenous immunoglobulin 20 g/day for 5 days and mycophenolate mofetil (MMF) 0.75 g/day. Oral methylprednisolone was then started at a dose of 24 mg/day and the dose was subsequently reduced by 4 mg each week to 2 mg/day. The VT storm was controlled completely, and the echocardiography showed LVEF increased to 33% and LVDd decreased to 60 mm (Fig. 1D). After discharge, amiodarone was gradually discontinued, methylprednisolone was reduced to 2 mg/day, sacubitril/valsartan was increased to 75 mg/day, and the other drugs continued to use. During follow-up, the patient had no symptoms of heart failure, arrythmia, and diplopia. ICD programming showed the VT no longer recurred (Fig. 2). Nine months after discharge, his last follow-up data showed remarkable recovery in LV systolic function (LVEF 52%) and normal CK level (38 U/L) (Fig. 1E).

## Discussion and conclusions

AMAs, the characteristic markers of primary biliary cirrhosis (PBC) [2], also were identified as myositis-related antibodies (MRAs) of IIM [3]. In the US, Johns Hopkins Myositis Center reported only 0.006% patients with myositis had positive AMAs [4]. On the contrary, the proportion in Asian countries is much higher. A study from Japan reported 11.3% myositis patients had positive AMAs and the incidence ratio of males to females varies from 9:15−1:6 [5]. Two Chinese patient cohorts reported the positive ratio varied from 2.5 –5% [6, 7]. In addition, it was identified that IMNM was the major histopathological finding in AMAs-positive IIM, which is consistent with the current report [6].

AMA-positive inflammatory myopathy usually has chronic course, characterized by muscle atrophy, histological granulomatous inflammation and cardiopulmonary involvement [5]. The cardiac involvement in AMA-related inflammatory myopathy includes arrhythmias, reduced ejection fraction, conduction abnormalities, cardiomyopathy, ventricular dilatations, myocarditis, mimicking cardiac sarcoidosis, cardiac arrest and so on [4, 8, 9]. The cardiac injuries in AMAs-positive myopathy is more prevalent than AMAs-negative IIMs [5, 6, 10] and usually precede muscle injuries [4], which could lead to the misdiagnosis of cardiomyopathy. Nevertheless, the mechanism of cardiac involvement in AMA-positive myopathy is unclear yet. It has been reported that the antibodies against cardiac myocytes exhibited a cytotoxic effect, damaging the mitochondrial of myocytes, which in turn disturbs the energy metabolism via inhibition of nucleotide transport [11, 12].

In our case, the patient received ICD therapy due to experienced repeated VT and severe reduced LVEF. However, ICD is not an etiological treatment, and the recurrent VT storm is difficult to control. Electrical storms were defined as three or more episodes of ventricular arrhythmias within 24 h [13]. In addition, patients who experienced electrical storm had a significantly higher risk of death, rehospitalization and anxiety [14]. Therefore, it is critical to identify and treat the primary disease to maintain the rhythm after cardioversion in the long term.

According to the recommendations, the diagnosis of inflammatory myopathy needs positive biopsy findings, and excluding muscular dystrophy, inclusion body myositis and sarcoid myopathy [15–17]. Our pathologist made a differential diagnosis of slices overdyed with different antibodies. Most importantly, our cardiologists made a relatively definitive diagnosis of the disease without high pathological specificity by combining the patient’s muscular atrophy with cardiomyopathy and repeated VT. As the inflammatory course played a pivotal role in the cardiac injury, steroid and immunosuppressive therapy could be effective [18, 19]. Eventually, after the combination of methylprednisolone, immunosuppressor, human immunoglobulin and sedative drugs, the VT storm was controlled and the cardiac remodeling was reversed.

Dilated cardiomyopathy is a heterogeneous disease with numerous etiologies, making differential diagnosis challenging. Therefore, the cardiac injuries would probably progress to an uncontrolled situation. In addition, the long-term survival rate for such patients is unclear yet. Meanwhile, the efficacy of the treatment may be individualized and heterogeneous. The data related to this disease needs to be further enriched. Thus, the patients need to be followed up closely in the future and the treatment plans should be made in advance for fear of recurrence of the VT storm.

In summary, early detection and timely treatment are essential for preventing the deterioration of the AMA-positive myopathy. AMA could be screened when discovering myopathies accompanied by unexplained cardiac symptoms. Conversely, cardiovascular disease should also be screened when AMA-positive myopathy is suspected.

## Data Availability

All relevant information is contained within the present manuscript.

## Abbreviations

VT: Ventricular tachycardia
ICD: Implantable cardioverter-defibrillator
AMA: Anti-mitochondrial antibody
HR: Heart rate
CK: Creatine kinase
CK-MB: Creatine kinase isoenzyme
CMR: Cardiac magnetic resonance
CTA: Computed tomography angiography
LVEF: Left ventricular ejection fraction
EMG: Electromyogram
ANA: Anti‐nuclear antibodies
RWMA: Regional wall motion abnormality
BNP: Brain natriuretic peptide
IIM: Idiopathic inflammatory myopathy
IMMN: Immune-mediated myositis necrotizing
MMF: Mycophenolate mofetil
PBC: Primary biliary cirrhosis
MRAs: Myositis-related antibodies
